# Vibronically Coupled and Thermally Tunable Broadband NIR Optical Response in 0D W^4+^‐Activated Cs_2_ZrCl_6_ Perovskite for Multifunctional NIR Spectroscopy Applications

**DOI:** 10.1002/advs.202511291

**Published:** 2025-08-13

**Authors:** Fangxue Chen, Yu Zha, Fanju Meng, Qiudong Duan, Jin Han, Yugeng Wen, Jianbei Qiu

**Affiliations:** ^1^ Faculty of Material Science and Engineering Kunming University of Science and Technology Kunming 650093 China; ^2^ Key Lab of Advanced Materials of Yunnan Province Kunming 650093 China; ^3^ Southwest United Graduate School Kunming 650092 China

**Keywords:** anti‐thermal quenching, broadband near‐infrared emission, HF gas sensing, NIR spectroscopy applications, vibronic coupling

## Abstract

Low‐dimensional halide perovskites are highly susceptible to thermal quenching (TQ) due to strong soft lattice nature. Currently, examples of thermally enhanced NIR luminescence in low‐dimensional materials are very scarce to the knowledge. Herein, the active role of vibronic coupling is manifested through thermal tunability of broadband NIR emission in 0D W^4+^‐activated Cs_2_ZrCl_6_, leading to anti‐TQ behavior ranging from 80 to 613 K. Interestingly, the internal quantum efficiency is dramatically boosted from 55.9% to 92.9% in Cs_2_ZrCl_6_: W^4+^, Ce^4+^ while retaining zero‐TQ luminescence between 303 and 423 K. Transient‐state spectroscopy reveal the distribution of thermally released charge carriers among the vibronically coupled *d*‐electronic states of W^4+^ ion is responsible for excellent thermal stability. Density functional theory calculations confirm that weak transient lattice distortion of isolated [WCl_6_]^2–^ octahedra in the excited state can combat TQ enabled by Franck–Condon vibronic coupling. Utilizing this thermal‐tolerant characteristic, both bandwidth‐ and lifetime‐based thermometers have been developed with low temperature uncertainties below 0.12 K. Moreover, NIR spectroscopy‐type sensor is presented for quantitative HF gas detection with concentration‐ and temperature‐dependent high sensing response and low detection limit. These findings may provide a vital insight into vibronic coupling‐assisted heat‐favorable NIR emissions in low‐dimensional materials for versatile applications.

## Graphical abstract

A vibronic coupling‐assisted way is provided for circumventing thermal quenching related to transient lattice distortion in 0D halide perovskites, and this study offers comprehending electron–lattice interactions in low‐dimensional host–W^4+^ dopant systems for the development of thermally tunable NIR spectroscopy applications, such as HF gas sensing and thermometry.

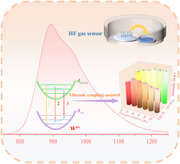

## 1 Introduction

Nowadays, broadband near‐infrared (NIR) phosphors play a key role in NIR spectroscopy applications such as nondestructive detection, medical diagnosis, optical communication, and phototherapy.^[^ ^1^, ^2^, ^3^, ^4^ ^]^ Various metal ions have been intensively studied as promising NIR activators for phosphor‐converted light‐emitting diodes (pc‐LEDs), including Cr^3+^, Ni^2+^, Sb^3+^, Mn^2+,^ and Eu^2+^.^[^ ^5^, ^6^, ^7^, ^8^, ^9^ ^]^ However, large emission bandwidth usually means strong electron‒phonon coupling (EPC), which is favorable for lattice relaxation upon photoexcitation, leading to obvious thermal quenching (TQ). The TQ issue can be greatly reduced in luminescent materials with large structural rigidity. For example, Cheng et al. reported anti‐TQ effect of Mn^2+^ green emission in a mineral structure‐inspired phosphor with dense framework in a very wide temperature range (303–463 K).^[^ ^10^ ^]^ Lim and co‐workers also reported anti‐TQ behavior in Cr^3+^‐doped spinel‐type phosphors with 125.53% of the initial photoluminescence (PL) intensity at 423 K.^[^ ^11^ ^]^ Xia's group has developed a chemical substitution strategy in a CaScAlSiO_6_ phosphor to enhance the structural rigidity for thermally stable Cr^3+^‐based broadband NIR luminescence.^[^ ^12^ ^]^ Therefore, structure rigidity has been considered as a key factor for realizing low TQ or even anti‐TQ behaviors in common phosphors.

Unlike 3D materials, low‐dimensional halide perovskites are highly susceptible to thermal quenching due to their soft lattice nature. In particular, 0D metal halides, featured with individual metal halide polyhedral or clusters, often undergo significant lattice distortion in the excited state upon photoexcitation, leading to serious energy loss at high temperatures. The reported vacancy‐ordered double perovskites Cs_2_XCl_6_ (X = Zr, Hf, and Te) have 0D electronic structure, resulting in [XCl_6_]^2−^ octahedra with more degrees of freedom.^[^ ^13^, ^14^, ^15^, ^16^ ^]^ The formation of self‐trapped exciton (STE) is promoted by such soft lattice, producing broadband emissions with strong TQ effects. Recently, both Mo^4+^ and W^4+^ ions have been introduced as NIR emission centers in the 800–1300 nm range, despite the origins of these emissions are still under debate and their temperature‐dependent luminescence behaviors are also case by case.^[^ ^17^, ^18^, ^19^, ^20^, ^21^ ^]^ Liu's group reported that STE is responsible for the NIR emissions of Cs_2_MoCl_6_ and Cs_2_WCl_6_ with thermal‐enhanced behaviors above room temperature.^[^ ^17^ ^]^ However, Kumar et al have ascribed the NIR emission to the Mo d‒d transitions instead of STE, and only found emission enhancement ranging from 250 to 300 K.^[^ ^18^ ^]^ Wu et al claimed that NIR emission of Cs_2_WCl_6_ nanocrystal originates from STE with obvious TQ phenomenon due to its low dimensionality and localized nature.^[^ ^19^ ^]^ Hence, further studies should be performed for better understanding the excited‐state mechanism of the temperature‐dependent NIR luminescence.

Usually, thermal‐induced emission loss can be compensated by negative thermal expansion, phonon assistance, energy transfer, crystal defect, and phosphor in glass.^[^ ^22^, ^23^, ^24^, ^25^, ^26^, ^27^, ^28^, ^29^, ^30^, ^31^, ^32^ ^]^ However, examples of thermally enhanced NIR luminescence in low‐dimensional halide perovskites are very scarce, due to strongly transient lattice distortion in the excited state. Hence, achieving anti‐TQ effects in low‐dimensional materials while maintaining high internal quantum efficiency (IQE) is still challenging. Herein, we first report the design strategies for realizing high quantum efficiency and thermally enhanced W^4+^ NIR emission in 0D Cs_2_ZrCl_6_ across the ultrawide temperature range (80–613 K). By systematic studies, we identify that vibronic coupling between the electronic transitions of [WCl_6_] octahedron and lattice vibrations can combat TQ originating from weak structural rigidity of [ZrCl_6_] octahedra network, and Ce^3+^ doping strategy also helps to overcome the energy‐gap law at elevated temperatures, thereby effectively mitigating the TQ effect. In addition, dual‐mode temperature sensors with low temperature uncertainties have been demonstrated, and temperature‐dependent gas sensing of toxic HF with low detection limit has also been presented based on its strong molecular absorption between 865 and 900 nm, demonstrating promising NIR spectroscopy applications.

## 2 Results and Discussion

The Cs_2_ZrCl_6_ (CZC), W^4+^‐doped CZC (abbreviated as CZC:W), and Ce^4+^, W^4+^‐codoped CZC (CZC:W/Ce) were synthesized via a facile hydrothermal method. According to the doping concentrations, for example, the corresponding samples can be named as CZC:7 W or CZC:7 W/7Ce under single or co‐doping conditions, respectively. The W^4+^ source comes from WCl_5_, and thus CeCl_3_ is introduced to further reduce W^5+^ to W^4+^ according to the following redox reaction:

(1) $$\mathrm{CeC}l_{3}\left(s\right)+\mathrm{WC}l_{5}\left(s\right)\to \mathrm{CeC}l_{4}\left(s\right)+\mathrm{WC}l_{4}\left(s\right)$$

**Figure 1:**
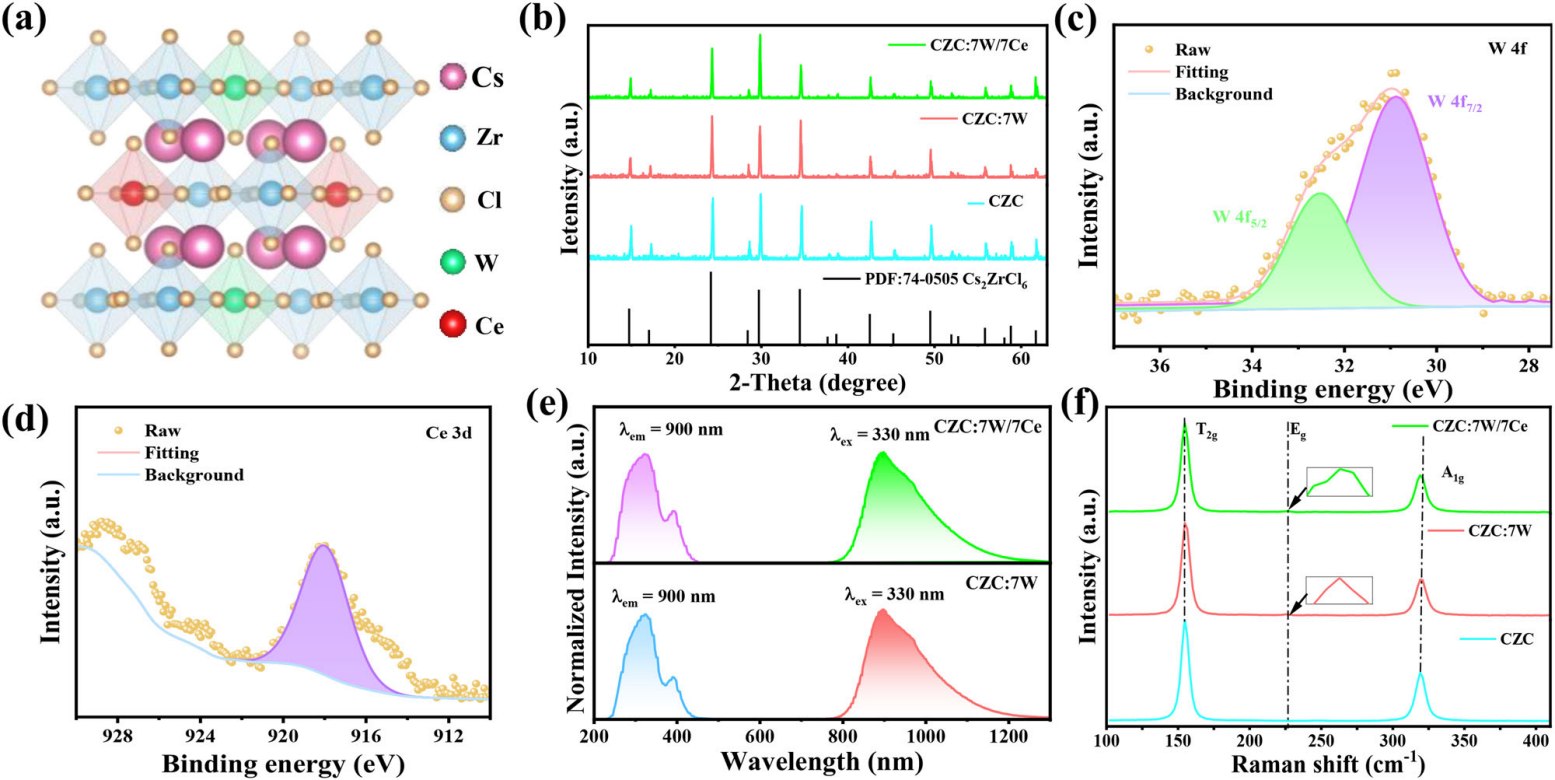
a) Crystal structure of CZC:W/Ce. b) Powder XRD patterns of CZC, CZC:W and CZC:W/Ce with doping concentration of 7%. c,d) XPS spectra of W 4f and Ce 3d of CZC:7 W/7Ce, respectively. e) PLE and PL spectra of CZC:7 W/7Ce. f) Raman spectra of CZC, CZC:7 W and CZC:7 W/7Ce.

As shown in **Figure** **1**a, the crystal structure of CZC:W/Ce is a typical vacancy‐ordered double perovskite 0D structure, where discrete [WCl_6_]^2−^ or [ZrCl_6_]^2−^ octahedra are isolated by Cs^+^ ions. After doping with W^4+^ and Ce^4+^, the X‐ray diffraction (XRD) patterns of these samples match well with the standard card of CZC (PDF#74‐0505), indicating high phase purity (Figure 1b). In addition, the high‐resolution X‐ray photoelectron spectroscopy (XPS) spectra of W 4f and Ce 3d confirm that both the corresponding oxidation states are +4 (Figure 1c,d; and Figure S1, Supporting Information).^[^ ^20^, ^33^ ^]^ The single intense peak in the Ce 3d spectra may be ascribed to shake‐up satellite peak of Ce^4+^ instead of Ce 3d_5/2_ and Ce 3d_3/2_ peaks, implying that the Ce^4+^ 3d electrons do not participate in the chemical bond formation that is strongly different from other reported cases. All the elements have been evidenced in XPS results (Figures S2 and S3, Supporting Information), and have been distributed homogeneously as shown in energy‐dispersive X‐ray analysis (Figure S4, Supporting Information). The actual doping content of W^4+^ and Ce^4+^ were determined from the induced coupled plasma optical emission spectrometry in Table S1 (Supporting Information). A broadband NIR emission centered at 900 nm can be found in CZC:7 W/7Ce upon 330 nm excitation, proving the successful introduction of W^4+^ ions (Figure 1e). The strong absorptions between 280 and 340 nm are associated with the d_xy_ to d_z_^2^ transitions of W^4+^ ions, with calculated optical band gaps ≈3.31 eV (Figure S5, Supporting Information).

The low phonon frequencies evidenced by Raman spectra indicate weak EPC effects in the as‐prepared samples (Figure 1f), which can reduce nonradiative losses according to the energy‐gap law, especially in the NIR range.^[^ ^2^, ^34^ ^]^ Three Raman modes correspond to the breath vibration of Zr─Cl bond (T_2g_), asymmetric stretching (E_g_), and symmetric stretching (A_1g_) of [ZrCl_6_]^2−^ and [WCl_6_]^2−^octahedra, respectively. Moreover, the very weak E_g_ modes after W substitution mean that Jahn‒Teller distortion can hardly occur in CZC:7 W and CZC:7 W/7Ce.^[^ ^21^ ^]^ Such Raman‐active vibronic coupling is likely a Franck–Condon process, which shows positive effects on broadband NIR emission as discussed below.^[^ ^35^ ^]^

**Figure 2:**
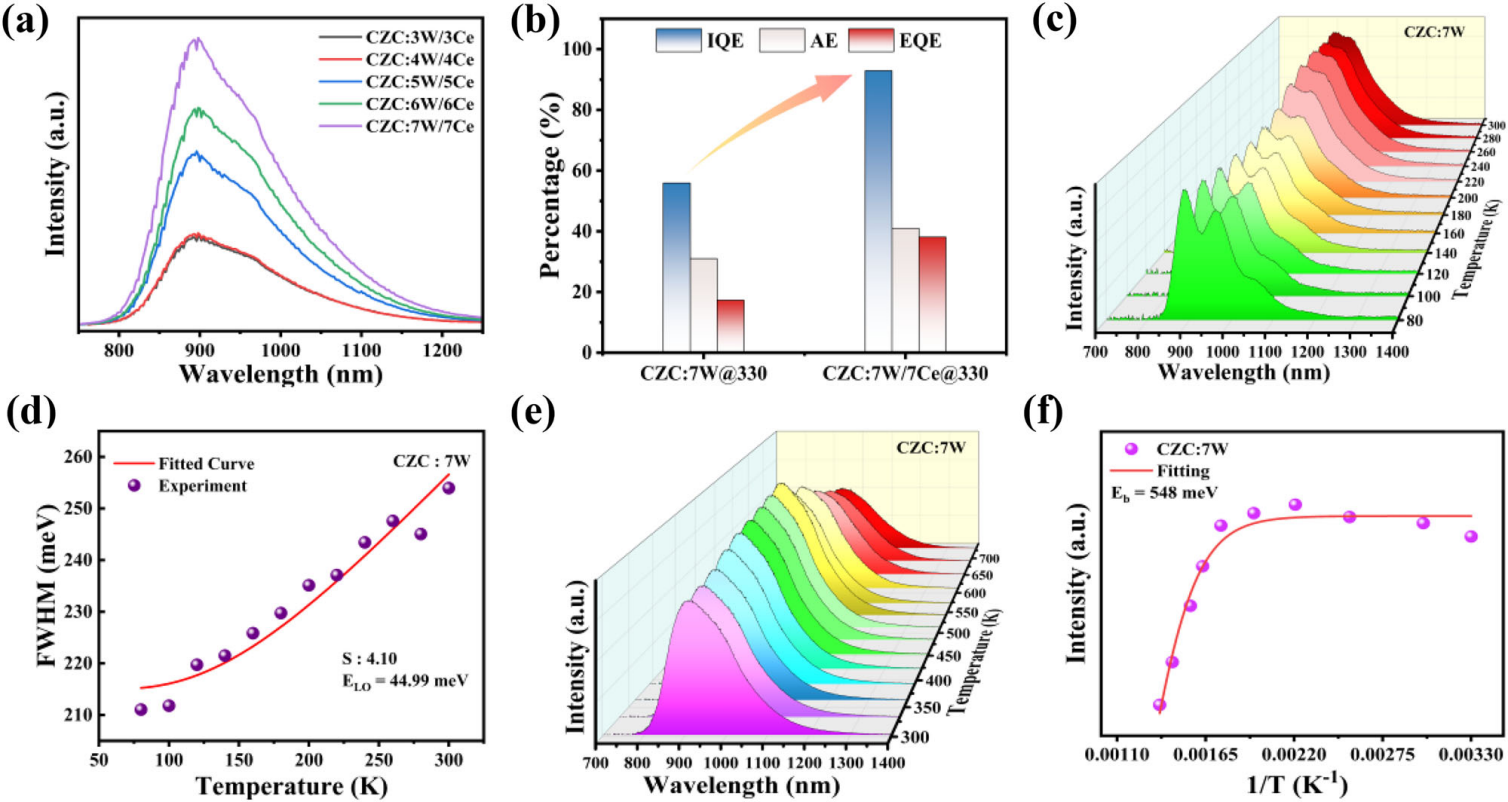
a) PL spectra of CZC:W/Ce with different doping concentrations. b) IQE, AE and EQE values of CZC:7 W and CZC:7 W/7Ce. c) Temperature‐dependent PL spectra of CZC:7 W under 330 nm excitation, ranging from 80 to 300 K. d) Fitting results of the Huang‐Rhys factor (*S*) and phonon energy (*E*_LO_) based on the relationships between FWHM and temperature for CZC:7 W under 330 nm excitation. e) Temperature‐dependent PL spectra of CZC:7 W under 330 nm excitation, ranging from 303 to 733 K. f) The integrated PL intensity versus 1/T and the fitting result of *E*_b_ for CZC:7 W.

The optimal concentrations of W^4+^ and Ce^4+^ ions are 7% according to the PL measurements (**Figure** **2**a; Figure S6, Supporting Information), and the following characterizations would be carried out based on this. As shown in Figure 2b and Figure S7 (Supporting Information), the best‐performing CZC:7 W/7Ce sample exhibits internal quantum efficiency (IQE) of 92.9% and absorption efficiency (AE) of 41% under 330 nm excitation, leading to a high external quantum efficiency (EQE) of 38.1%, among the best reported values of reported NIR phosphors (Figure S8, Supporting Information). In contrast, CZC:7 W sample shows moderate IQE of 55.9% and AE of 31%, indicating the promoting effect of Ce doping. Low‐temperature PL measurements (80–300 K) were conducted for CZC:7 W and CZC:7 W/7Ce (Figure 2c; Figure S9, Supporting Information), the integrated PL intensities increase with the enhanced temperatures, due to temperature‐facilitated formation of vibronically coupled d‒d transitions of W^4+^. The Huang–Rhys factor *S* has been calculated by analyzing the variation of full width at half‐maximum (FWHM) as a function of temperature using the following equation:

(2) $$FWHM\left(T\right)=2.36\sqrt{S}\hbar \omega_{\mathrm{phonon}}\sqrt{\coth\frac{\hbar \omega_{\mathrm{phonon}}}{2k_{B}T}}$$

where ℏω_phonon_ is the phonon energy, *K*_B_ is the Boltzmann constant, and *S* is the electron–phonon coupling parameter. The phonon energy of CZC:7 W is 45 meV, which is consistent with the Raman results, further confirming the accuracy of calculation analysis based on the above equation. The fitted *S* values are 4.1 and 4.58 for CZC:7 W and CZC:7 W/7Ce, respectively, suggesting weak electron–phonon coupling (Figure 2d; Figure S10, Supporting Information).^[^ ^36^, ^37^ ^]^ The low values of *S* mean that NIR emissions come from the W d–d transitions, in line with the recent results of Mo^4+^, different from STE mechanism of W^4+^ accepted by other reports.^[^ ^17^, ^18^, ^19^, ^20^, ^21^ ^]^

Impressively, the anti‐TQ behavior has also been observed in high‐temperature regions for CZC:7 W. As shown in Figure 2e, the emission intensities could be boosted with rising temperatures from 303 to 613 K, reaching 121%@543 K and 102%@613 K of the initial intensity at 303 K, respectively. Regarding CZC:7 W/7Ce, there is almost no change of luminescence intensity in the range of 303–423 K (Figure S11, Supporting Information), and the integrated PL intensities at 543 K can still maintain 91% of the initial intensity at room temperature, demonstrating excellent thermal stability above room temperature (Figure S12 and Table S2, Supporting Information). The temperature dependence of the integrated PL intensity can be fitted by the Arrhenius formula:

(3) $$I\left(T\right)=\frac{I_{0}}{1+A\exp\left(\;\frac{E_{b}}{K_{B}T}\right)}$$

where *I*_0_ and *I(T)* are the integrated PL intensity at 0 and T (K), respectively, A is a constant, *E*_b_ represents exciton binding energy, and *K*_B_ is the Boltzmann constant. The fitting results show that *E*_b_ values are determined to be 548 and 321 meV for CZC:7 W and CZC:7 W/7Ce, respectively (Figure 2f; Figure S13, Supporting Information). The much larger *E*_b_ means that the dissociation of excitons can be effectively avoided at high temperatures, which is beneficial for stable NIR emissions.^[^ ^38^ ^]^

**Figure 3:**
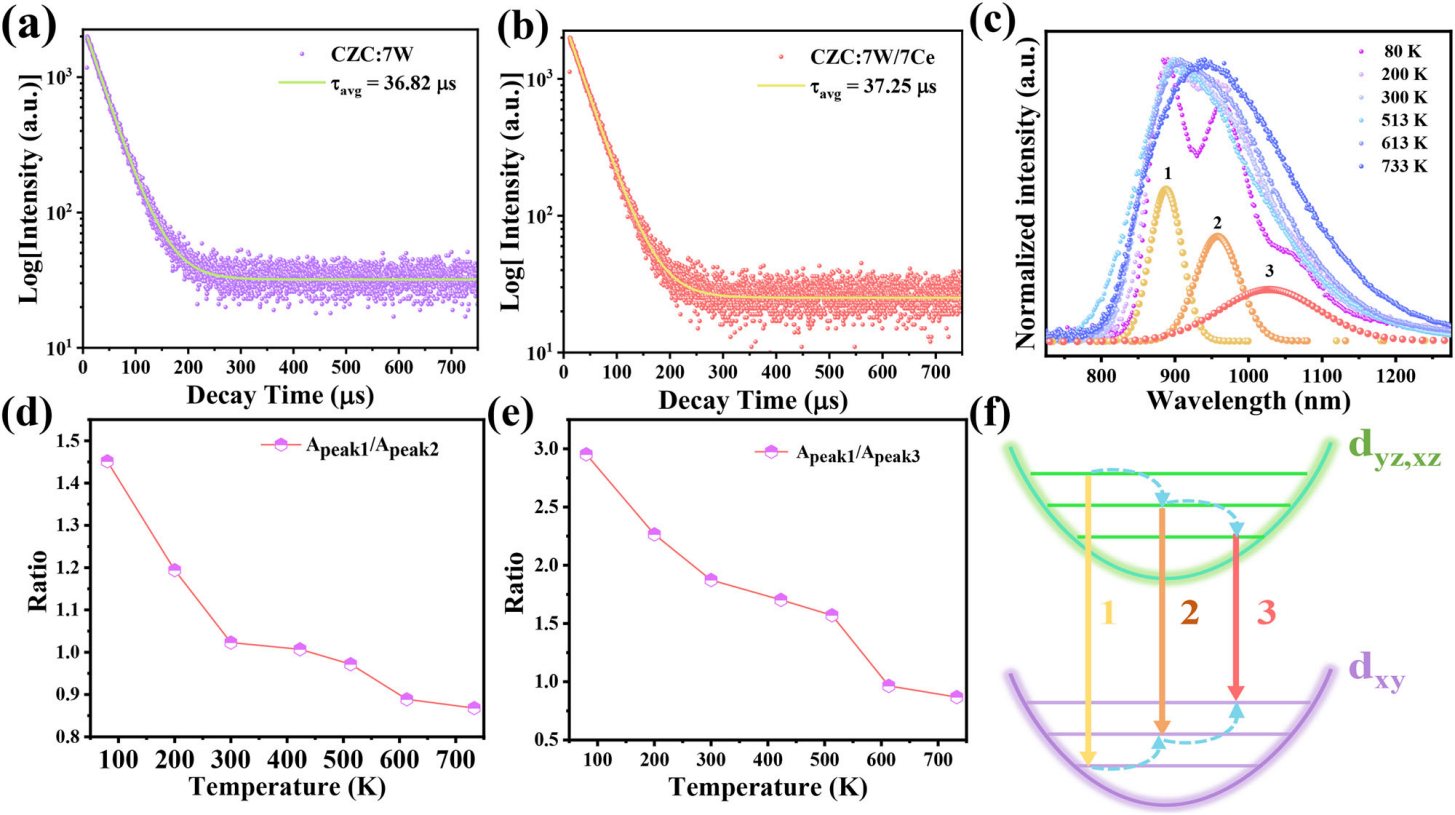
a, b) PL decay times of CZC:7 W and CZC:7 W/7Ce at 900 nm under 330 nm excitation. c) Normalized emission spectra fitted with a Gaussian function. d, e) The integrated intensity ratios of A_peak1_ and A_peak2_, A_peak1_ and A_peak3_ between 80 and 733 K, respectively. f) Diagram of electron population redistribution in sub‐levels of W^4+^ due to vibronically coupled d‒d transitions.

The excited‐state dynamics results show that CZC:7 W/7Ce has a longer lifetime than that of CZC:7 W (**Figure** **3**a,b), indicating that the main contribution of quantum efficiencies comes from the improved crystallization due to reduced lattice defects and nonradiative recombination rate after Ce doping.^[^ ^39^ ^]^ The PL spectra can be fitted using a Gaussian function, with three obvious luminescent peaks centered at 900, 958, and 1028 nm, respectively, (Figure 3c). Three sub‐peaks correspond to the sub‐levels between the d–d transitions of 1, 2, and 3, which quantifies the electron population redistribution at different temperatures. As shown in Figure 3d, the integrated intensity ratio between the peak 1 (A_peak1_) and the peak 2 (A_peak2_) decrease from 1.45 at 80 K to 0.87 at 733 K, indicating the redistribution of the d‒d transition probability at 1 and 2 sub‐levels. More apparently, the integrated intensity ratio between the peak 1 (A_peak1_) and the peak 3 (A_peak3_) drops dramatically from 2.95 to 0.87 at the same temperature range, demonstrating a phonon‐assisted NIR emissions at the longest wavelength (Figure 3e). Based on the PL results, the d–d transition of W^4+^ ion is probably a Franck–Condon process, where the vibronic coupling between electronic transitions of W^4+^ and lattice vibrations is responsible for thermally manipulating the transition probability of these sub‐levels (Figure 3f).^[^ ^35^ ^]^ Thus, with the positive effects of vibronic coupling, the intrinsic problems restricted by energy‐gap law can be mitigated, which facilitates the improvement of thermal stability.

**Figure 4:**
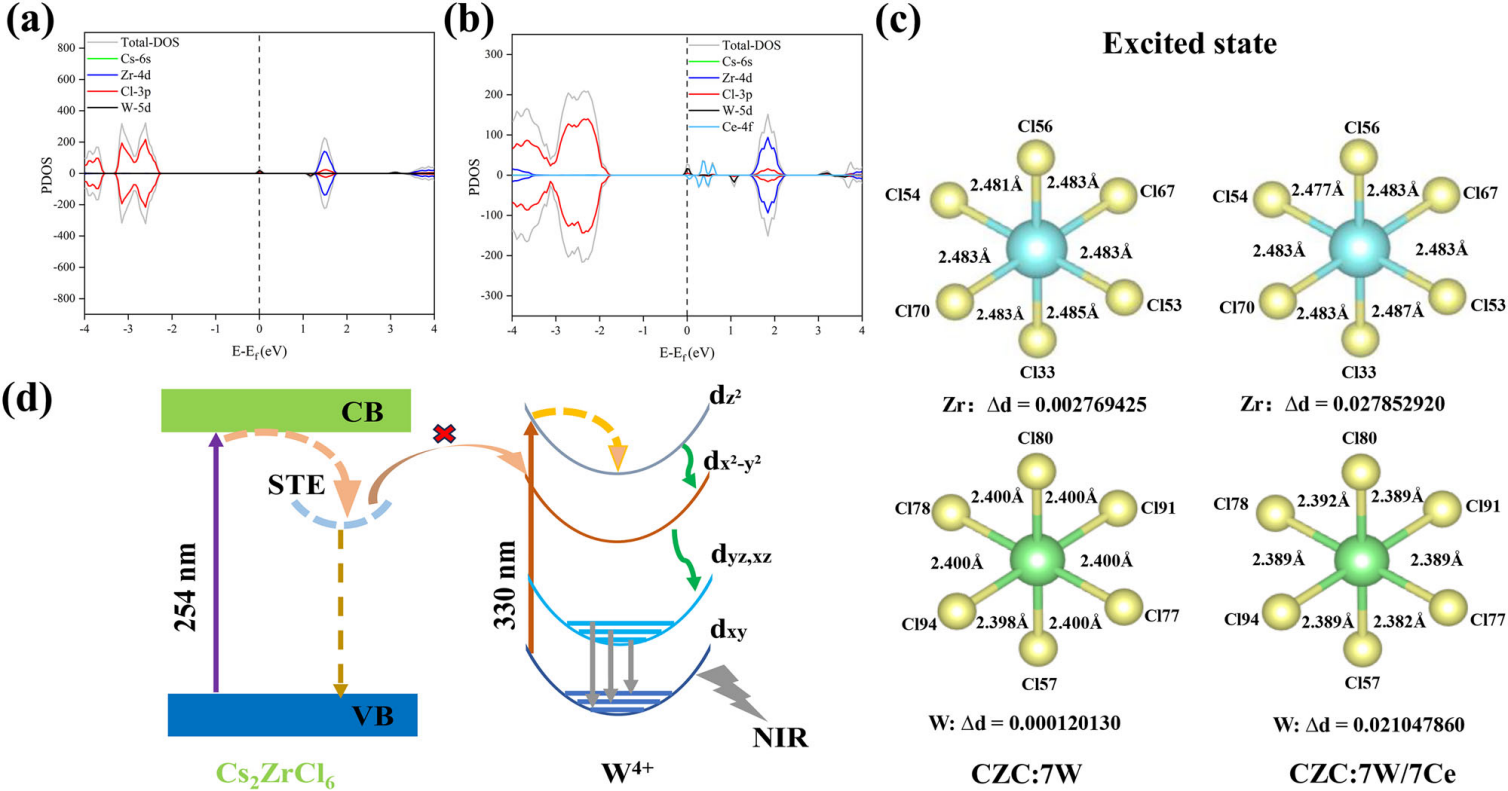
a,b) DOS of CZC:7 W and CZC:7 W/7Ce, respectively. c) The calculated excited state distortion of [ZrCl_6_]^2−^ and [WCl_6_]^2−^ octahedra in CZC:7 W and CZC:7 W/7Ce, respectively. d) Schematic energy diagram and NIR luminescent mechanism of W^4+^ dopant in CZC host.

The electronic band structure and density of state (DOS) were calculated using first‐principles‐based density functional theory. As shown in **Figure** **4**a,b, the hybridization of W 5d and Cl 3p orbitals leads to the introduction of midgap states responsible for the d‒d transitions. The band structure of CZC:7 W shows semiconducting behavior, while both metallic and semiconducting characteristics can be found in the spin‐up and spin‐down modes for CZC:7 W/7Ce (Figure S14, Supporting Information). The lattice distortion can be semi‐quantitatively characterized by the bond length quadratic elongation *λ*_oct_ and the deviation *Δ*_d_ (Tables S3–S5, Supporting Information). In the excited state of CZC:7 W, the [ZrCl_6_]^2−^ octahedra show much stronger Jahn‐Teller distortions compared with [WCl_6_]^2−^ octahedra, as evidenced by one order of magnitude larger *Δ*_d_ in Figure 3c. That means electron–lattice interactions are very weak for maintaining good structural rigidity in isolated [WCl_6_]^2−^ octahedra, independently of strong transient lattice distortion in [ZrCl_6_]^2−^ octahedra. As calculated by the energy transfer efficiency (*η*) from STE of [ZrCl_6_]^2−^ to [WCl_6_]^2−^ emitting center (Table S6 and Figure S15, Supporting Information), the *η* value is no more than 10% at different temperatures, suggesting the weak interaction between them. The calculation results in Table S7 (Supporting Information) also support very weak distortions of [CeCl_6_]^2‒^ octahedra, meaning that the IQE enhancement is mainly ascribed to the reduced non‐radiative recombination rate rather than increased radiative recombination rate. As presented in Figure 4d, such strong excited‐state distortion of [ZrCl_6_]^2−^ octahedra can easily form STE state due to soft lattice nature, leading to vibronic coupling‐induced TQ at high temperatures. In contrast, there is weak interplay between [ZrCl_6_]^2−^ and [WCl_6_]^2−^ octahedra, so that W^4+^ ion can be self‐excited among the d_xy_ to d_z_^2^ transitions at 330 nm. Moreover, vibronic coupling plays the positive effects, rather than a negative role, on the d_yz_,_xz_ to d_xy_ transitions at elevated temperatures for circumventing thermal quenching in conventional 0D halide perovskites.

**Figure 5:**
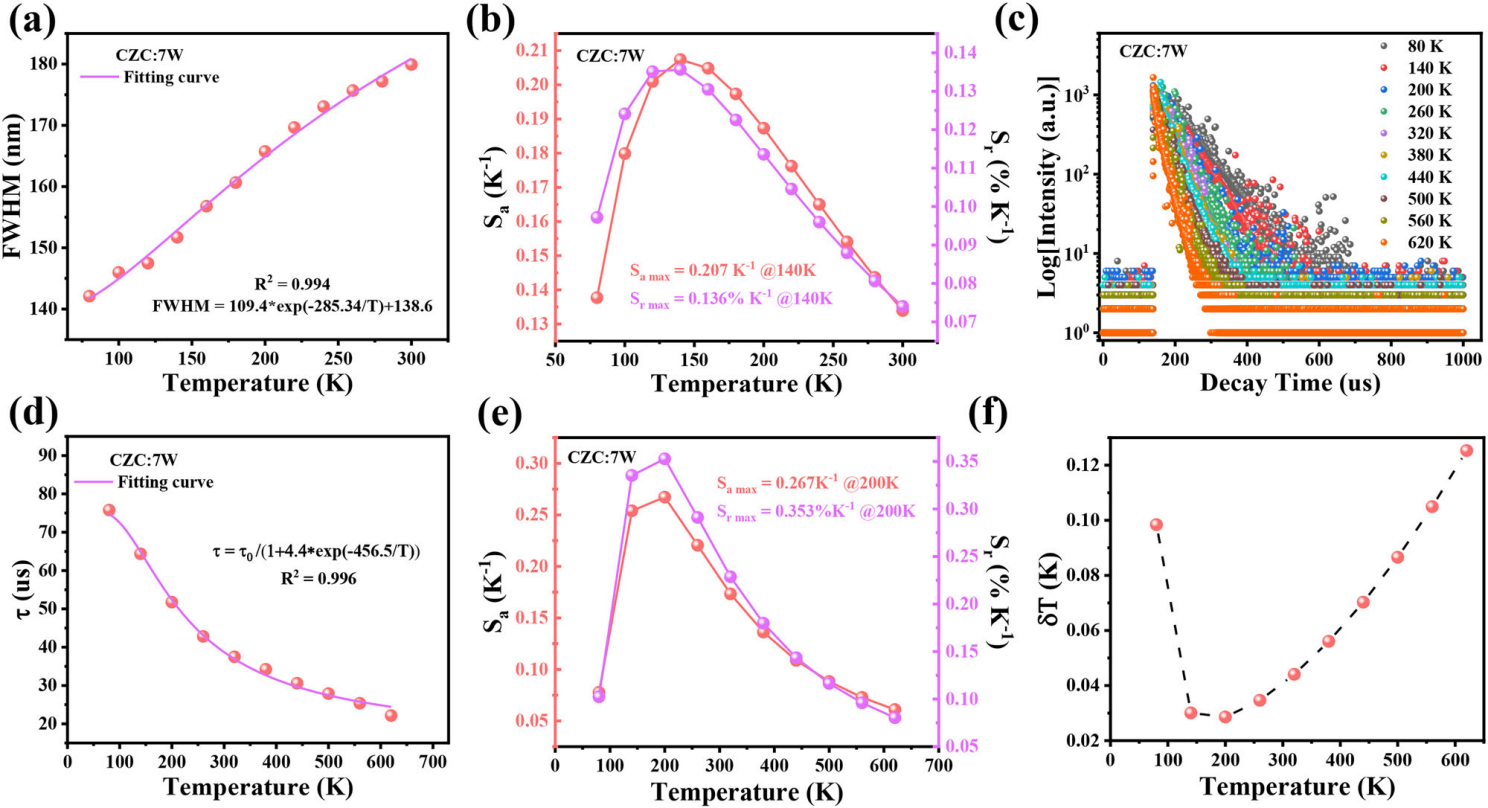
a) Temperature‐dependent bandwidth of CZC:7 W and corresponding fitting curve from 80 to 300 K. b) Calculated *S*_r_ and *S*_a_ values in the 80–300 K range. c, d) PL decay lifetimes of CZC:7 W at 900 nm under 330 nm excitation and corresponding fitting curve from 80 to 620 K. e) Calculated *S*_r_ and *S*_a_ values in the 80–620 K range. f) Calculated *δT* values in the 80–620 K range.

Thanks to the temperature‐dependent luminescent behaviors ranging from 80 to 620 K, both luminescence bandwidth‐ and lifetime‐mode thermometry methods can be applied to CZC:7 W and CZC:7 W/7Ce.^[^ ^40^ ^]^ As shown in **Figures** **5**a and S16 (Supporting Information), spectral bandwidth broadens homogeneously with the increased temperature, serving as good temperature sensors with the maximum relative sensitivity (*S*_r_) of 0.136% K^−1^ and absolute sensitivity (*S*_a_) of 0.207 K^−1^ at 140 K (Figure 5b). To confirm the practicality and accuracy of our material system for temperature sensing, extra experiments are performed as shown in Figure S17 (Supporting Information). As time goes, autoclave is cooled down with unknown temperature. By virtue of temperature‐dependent luminescent behaviors of material, the real‐time temperature of autoclave can be checked based on the fitting results in Figure S16a (Supporting Information). Meantime, infrared thermal imager is used to compare the temperature, and the testing results are in consistence with each other. In addition, the emission lifetime also shows high temperature dependence. The PL lifetimes of the peak 1 monitored at 900 nm decrease from 80 to 620 K (Figure 5c; Table S8, Supporting Information), which is favorable for lifetime‐based fluorescence thermometry. The *S*_r_ value can be calculated according to the following equation:^[^ ^26^, ^41^ ^]^

(4) $$S_{r}=\left|\frac{1}{\tau }\frac{d\tau }{dT}\right|\times 100\%$$

where *τ* is the lifetime at different temperatures, and *dτ/dT* is the relative change of lifetime per unit temperature change. Based on the decay fitting results of CZC:7 W (Figure 5d), the optimal *S*_r_ of 0.353% K^−1^ and *S*_a_ of 0.267 K^−1^ at 200 K can be obtained (Figure 5e). The temperature uncertainty (*δT*) is an important parameter for assessing the temperature measurement accuracy, which can be calculated as follows:

(5) $$\delta T=\frac{1}{S_{r}}\frac{\delta \tau }{\tau }$$

where *S*_r_ is the relative sensitivity and *δτ/τ* is the relative uncertainty in lifetime measurements. As shown in Figure 5f, all the *δT* values are well below 0.12 K from 80 to 620 K, demonstrating the potential for high‐precision temperature measurement applications.

**Figure 6:**
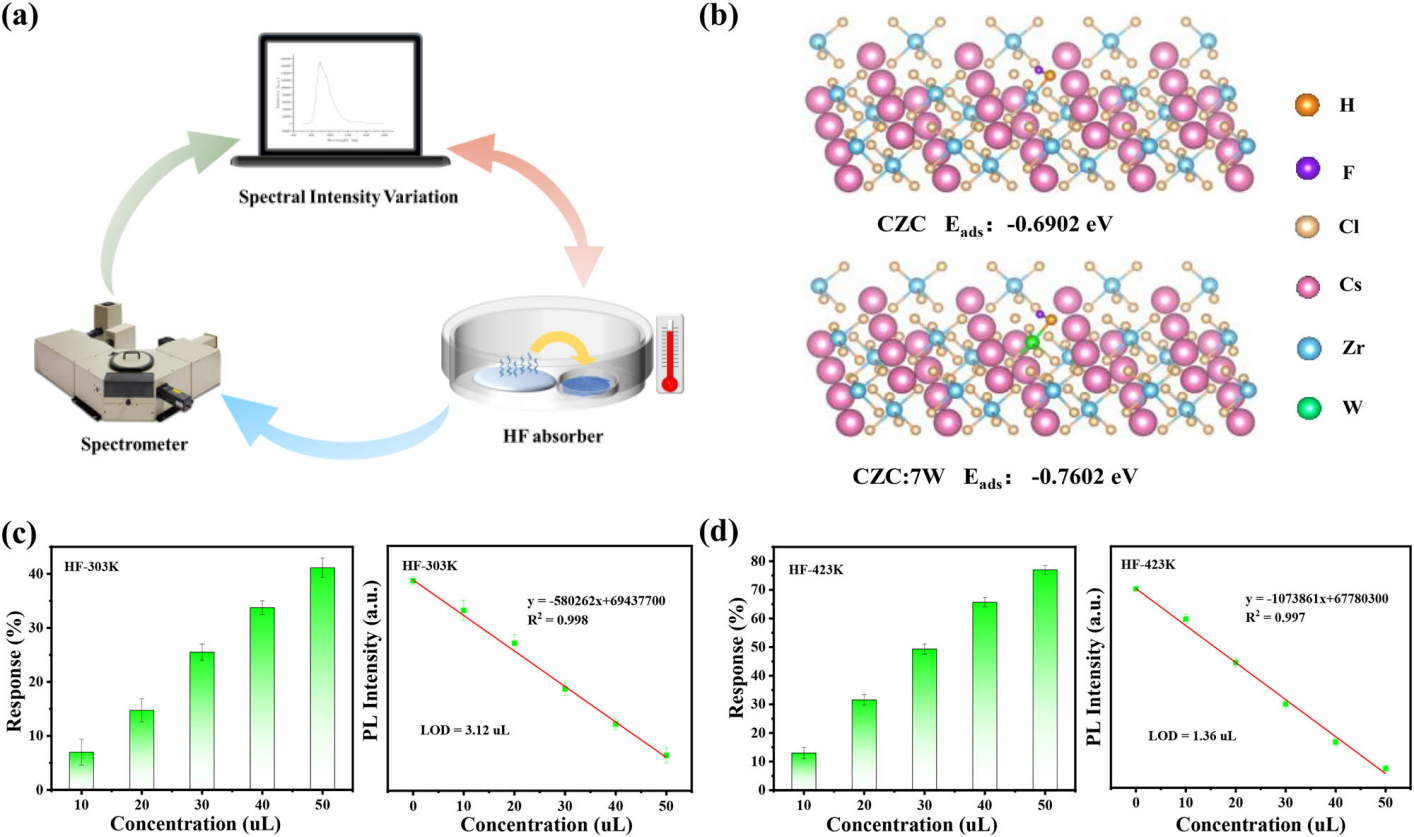
a) The setup of NIR spectroscopy‐type sensor for HF gas detection. b) The calculated HF adsorption energy in CZC:7 W and pure CZC. c, d) The response and LOD values under different HF concentrations at 303 and 423 K in CZC:7 W/7Ce, respectively. The error bars represent the standard deviation of both response and LOD values under 5 repeated measurements.

W‐based phosphors can also be as fluorescent probes for toxic HF detection, and the setup of NIR spectroscopy‐type gas sensor is presented in **Figure** **6**a. The surface of samples can adsorb HF gas under different temperatures, and then spectrometer is used to check the PL intensity variation before and after HF absorption. CZC:7 W and CZC:7 W/7Ce take advantage of two abilities for HF sensing: one is that HF has strong molecular absorption between 865 and 900 nm, well overlapped with intense NIR emission of W^4+^, thus leading to obvious NIR intensity variation for high sensitivity. Another is the enhanced HF adsorption capacities of W^4+^ ion in the CZC host. As shown in Figure 6b, the adsorption energy in CZC:7 W is lower than that of pure CZC, with calculated values of −0.7602 and −0.6902 eV, respectively (Table S9, Supporting Information). It is clear that the introduction of W is more energetically favorable for adsorbing HF molecules. To ensure the accuracy of sensing experiment, CZC:7 W/7Ce was chosen as the adsorbent due to its zero‐TQ characteristic, ensuring that the NIR intensity changes come only from HF adsorption. According to the reported definition equations, the response and detection limit (LOD) values are concentration‐ and temperature‐dependent.^[^ ^42^ ^]^ The response is defined as (I_sample_ ‒ I_HF_)/ I_sample_, where I_sample_ and I_HF_ are PL intensities before and after HF adsorption, respectively. LOD is calculated using *3σ/S*, where *σ* represents the standard derivation of samples and *S* denotes the slope of the calibration curve. The sensors yield the response values of ≈41% and 77% under the HF concentration of 50 µL, and LOD values of 3.12 and 1.36 µL at 303 and 423 K, respectively (Figure 6c,d). Both the two optical response metrics are sensitive to temperature (Figure S18, Supporting Information), indicating the outstanding analytical performance for quantitative HF gas detection.

## 3 Conclusion

An effective way of circumventing thermal quenching related to transient lattice distortion in 0D halide perovskites has been developed in this work. The heat‐tunable broadband NIR luminescence in both low‐ and high‐temperature regions (80–613 K) in W^4+^‐0D Cs_2_ZrCl_6_ is realized with boosted NIR emission quantum yield of 92.9%. The systematic studies reveal that vibronic coupling plays the positive effects on the *d*–*d* transitions of W^4+^ ions, including weakening the Jahn‐Teller distortion in the excited state, freeing the energy‐gap law and facilitating electron population redistribution at elevated temperatures. The isolation of [ZrCl_6_]^2−^ host and [WCl_6_]^2−^ dopant is also beneficial for anti‐ or zero‐thermal quenching NIR emissions from W^4+^ ions instead of self‐trapped excitons. By virtue of outstanding cold‐ and heat‐resistant NIR optical properties, bandwidth‐ and lifetime‐mode temperature sensors have been demonstrated, with low temperature uncertainties for potential high‐precision temperature measurement applications. Moreover, NIR spectroscopy‐type gas sensing of toxic HF with low detection limit and high response has also been presented for quantitative HF gas detection. The findings can not only achieve thermally enhanced broadband NIR emissions in 0D halide perovskites, but also allow comprehending electron–lattice interactions in low‐dimensional host–W^4+^ dopant systems toward versatile temperature‐dependent NIR spectroscopy applications.

## Conflict of Interest

The authors declare no conflict of interest.

## Supporting information



Supporting Information

## Data Availability

The data that support the findings of this study are available on request from the corresponding author. The data are not publicly available due to privacy or ethical restrictions.
