# Supplementary material for: Vibronically Coupled and Thermally Tunable Broadband NIR Optical Response in 0D W4+‐Activated Cs2ZrCl6 Perovskite for Multifunctional NIR Spectroscopy Applications
Source: Adv Sci (Weinh). 2025 Aug 13;12(42):e11291. doi: 10.1002/advs.202511291 (PMC12622407; doi:10.1002/advs.202511291)
Supplement: Supplementary file 1 — Supporting Information [file ADVS-12-e11291-s001.docx]

**Supporting Information**

**Vibronically Coupled and Thermally Tunable Broadband NIR Optical Response in Zero-Dimensional W^4+^-Activated Cs_2_ZrCl_6_ Perovskite for Multifunctional NIR Spectroscopy Applications**

*Fangxue Chen, Yu Zha, Fanju Meng, Qiudong Duan, Jin Han, Yugeng Wen,* and Jianbei Qiu**

_________________________________

F. Chen, Y. Zha, F. Meng, Q. Duan, J. Han, Y. Wen, J. Qiu

Faculty of Material Science and Engineering, Kunming University of Science and Technology, Kunming 650093, China

Key Lab of Advanced Materials of Yunnan Province, Kunming 650093, China

E-mail: [wenyg@kust.edu.cn](mailto:wenyg@kust.edu.cn); [qiu@kust.edu.cn](mailto:qiu@kust.edu.cn)

J. Qiu

Southwest United Graduate School, Kunming 650092, China

**Experimental Section**

**Materials**: Cesium chloride (CsCl, 99.9%, Aladdin), Zirconium chloride (ZrCl_4_, 99.9%, Aladdin), Cerium chloride hexahydrate (CeCl_3_·6H_2_O, 99.99%, Aladdin), Tungsten pentachloride (WCl_5_, 99%, Macklin), and hydrochloric acid (HCl, 37wt% in H_2_O, Aladdin). All materials and chemicals were used without further purification.

**Synthesis**: 2 mmol CsCl, 1–x mmol ZrCl_4_, x mmol CeCl_3_·6H_2_O and x mmol WCl_5_ were mixed in 10 mL HCl in a 25 mL Teflon liner. The mixture solution was heated at 180 ℃ for 12h in a stainless-steel Parr autoclave at a rate of 9 ℃/min, and cooled down to room temperature at a rate of 1 ℃/min. The precipitated products were then filtered out, washed with anhydrous ethanol, and vacuum dried.

**Characterization**: The powder XRD patterns were identified using a laboratory powder XRD system at a scanning rate of 2° min^-1^ in the 2*θ* range from 5° to 90°, with Cu Kα radiation (λ=0.15418 nm) at 40 kV and 40 mA. The morphological images of the samples were recorded by SEM (ZEISS Sigma 300, Germany), and energy-dispersive spectrometry (EDS) spectroscopy on a ZEISS Sigma 300 scanning electron microscope operated at 15 kV. Absorption spectra were recorded using UV-3600 (SPC, Japan). The photoluminescence was investigated using a Pro-FL spectrophotometer (F-7000, Hitachi, Japan). The photoluminescence quantum yields (PLQYs) were obtained directly by an absolute PL quantum yield measurement system (Hamamatsu Quantaurus-QY). The excitation source is a high-intensity xenon lamp. The excitation wavelength can be changed correspondingly, and the step increments and integration time were 1 nm and 0.5 s per data point, respectively. An integrating sphere was mounted on the spectrofluorometer with the entrance and exit ports set in 90° geometry. The sample was located in the center of the integrating sphere. All the recorded spectroscopic data were corrected for the spectral responses of both the integrating sphere and the spectrofluorometer. The responses of the detecting systems in photon flux were determined using a calibrated tungsten lamp. The experimental wavelength resolution is ±0.1 nm. The temperature-dependent and room temperature down-shifting photoluminescence excitation and emission spectra were recorded by a spectrofluorometer with a temperature controller (Edinburgh FLSP-920). PL decay dynamics (TRPL, time-correlated single photon counting) were measured using Edinburgh FLS980 Instrument. The elemental composition was determined using X-ray photoelectron spectroscopy (XPS, ESCALAB 250Xi, Thermo Scientific Inc., USA) with a monochromatic Al Kα source. There is no any cleaning step for the sample before testing. An appropriate amount of sample was pressed onto the sample plate in the chamber. The pressure of the chamber is less than 2.0 × 10^-7^, the spot size is 400 μm, the working voltage is 12 kV, and the filament current is 6 mA. The full spectrum scanning energy is 150 eV with a step size of 1 eV, and the narrow spectrum scanning energy is 50 eV with a step size of 0.1 eV. Raman spectra were obtained using a 532 nm wavelength laser on a LabRAM HR Evolution Raman spectrometer (HORIBA, France).

**Theoretical calculations:** The density functional theory (DFT) calculations were carried out in the Vienna Ab initio Simulation Package (VASP) based on the generalized gradient approximation (GGA) using the Perdew-Burke-Ernzerhof (PBE) formulation. The projected augmented wave (PAW) potentials were chosen to describe the ionic cores and take valence electrons into account using a plane wave basis set with a kinetic energy cutoff of 450 eV. Partial occupancies of the Kohn−Sham orbitals were allowed using the Gaussian smearing method and a width of 0.05 eV. For the optimization of both geometry and lattice size, the Brillouin zone integration was performed with 2×2×1 *Γ*-centered *k*-point sampling. The self-consistent calculations applied a convergence energy threshold of 10^-5^ eV. The equilibrium geometries and lattice constants were optimized with maximum stress on each atom within 0.02 eV Å^-1^. The weak interaction was described by DFT+D3 method using empirical correction in Grimme’s scheme. Spin polarization method was adopted to describe the magnetic system.^[1–8]^ The 15 Å vacuum layer was normally added to the surface to eliminate the artificial interactions between periodic images. The adsorption energy was calculated as: E_ads_ = E(*adsorbent) - E(*) - E(adsorbent). E(*adsorbent), E(*) and E(adsorbent) represent the total energy of * adsorbent, * and adsorbent molecule, respectively.

The distortion can be semi-quantitatively characterized by the bond length quadratic elongation *λ_oct_* and the deviations *Δ_d_* of the three trans Cl-W-Cl bond angles from 180°, according to Equations 1 and 2:

$\text{λ}_{\text{oct}}\text{ }\text{=}\frac{1}{6}\sum_{i=1}^{6} \left[ \frac{\left( d_{i}-d_{0} \right)}{d_{0}} \right]^{2}$ (1)

where *d_i_* is the W-Cl bond length, *d_0_* is the mean W-Cl bond distance. And

$\Delta_{d}=\frac{|d_{W-Cl1}+d_{W-Cl3}|}{|\cos\alpha_{Cl1-W-Cl3}|}+\frac{|d_{W-Cl2}+d_{W-Cl4}|}{|\cos\alpha_{Cl2-W-Cl4}|}+\frac{|d_{W-Cl5}+d_{W-Cl6}|}{|\cos\alpha_{Cl5-W-Cl6}|}$ (2)

The detailed values can be seen in Tables S3‒S5 and S7.

**Figure S1**. XPS spectra of Ce 3d of CZC:7W/7Ce by testing twice.

**Figure S2**. XPS spectra of CZC:7W.

**Figure S3**. XPS spectra of CZC:7W/7Ce.

**Figure S4**. SEM image and EDS distribution of the elements in CZC:7W/7Ce.

**Figure S5**. Absorption spectra of CZC:7W and CZC:7W/7Ce, and Tauc plot calculations based on the absorption spectra for CZC:7W and CZC:7W/7Ce, respectively.

**Figure S6**. PL spectra of CZC:W with different doping concentrations.

**Figure S7**. IQE and AE values measured for CZC:7W and CZC:7W/7Ce under 330 nm excitation, respectively.

**Figure S8**. Comparison of the IQE values of CZC:7W and CZC:7W/7Ce with those reported in the literature.


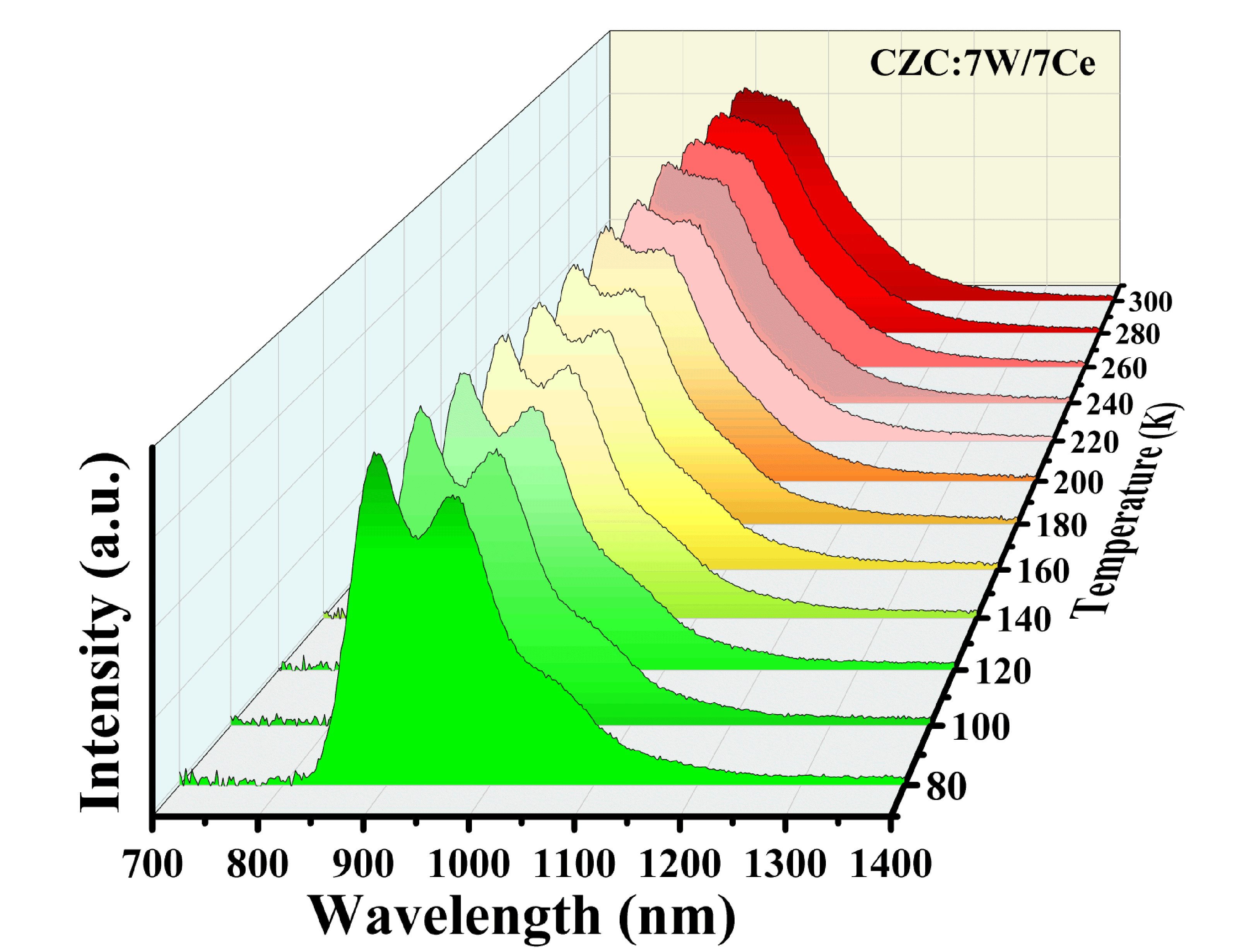


**Figure S9**. Temperature-dependent PL spectra of CZC:7W/7Ce under 330 nm excitation, ranging from 80 to 300 K.

**Figure S10**. Fitting results of the Huang-Rhys factor (*S*) and phonon energy (*E_LO_*) based on the relationships between FWHM and temperature for CZC:7W/7Ce under 330 nm excitation.


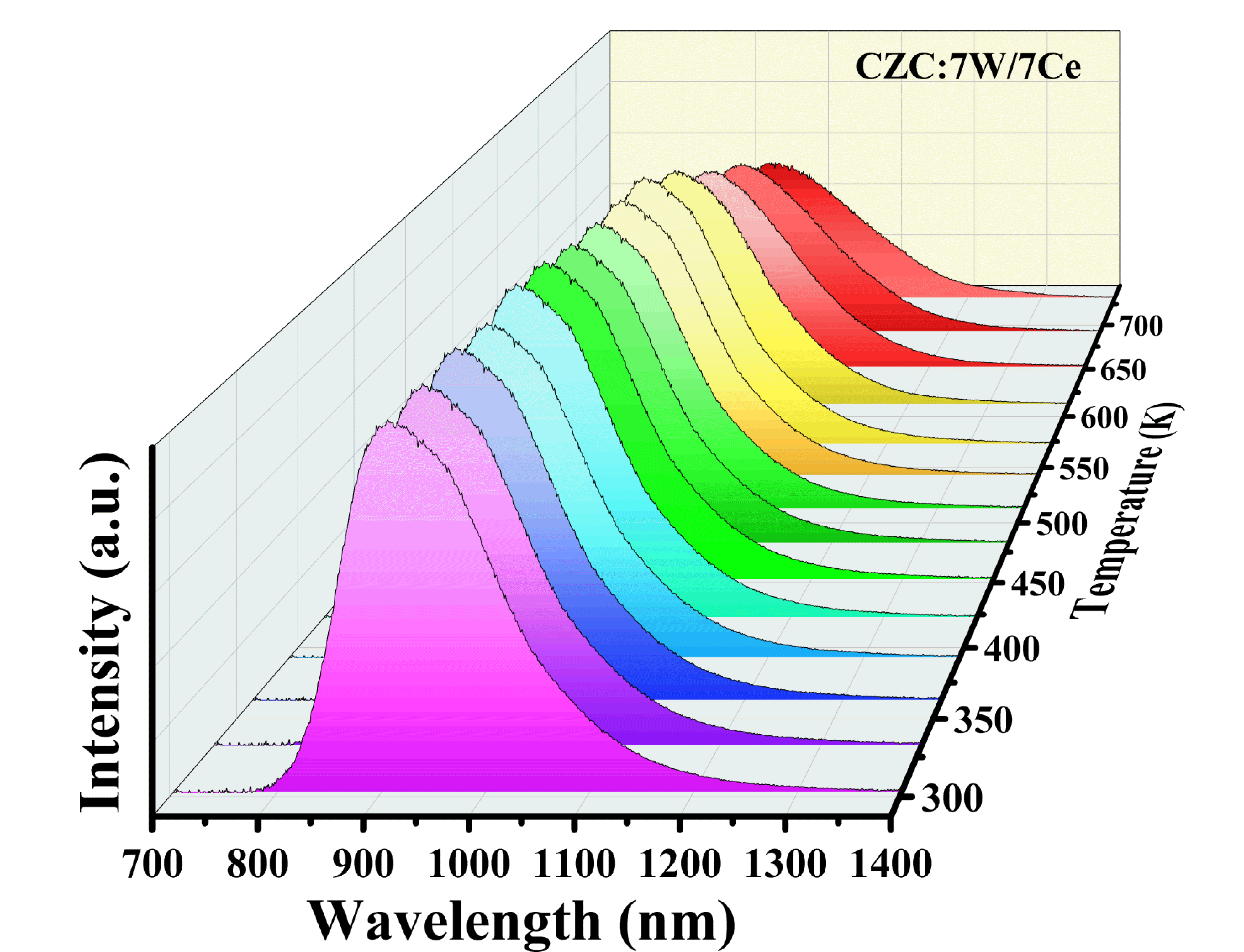


**Figure S11**. Temperature-dependent PL spectra of CZC:7W/7Ce under 330 nm excitation, ranging from 303 to 733 K.

**Figure S12**. Comparison of thermal stability of CZC:7W and CZC:7W/7Ce with those reported in the literature.


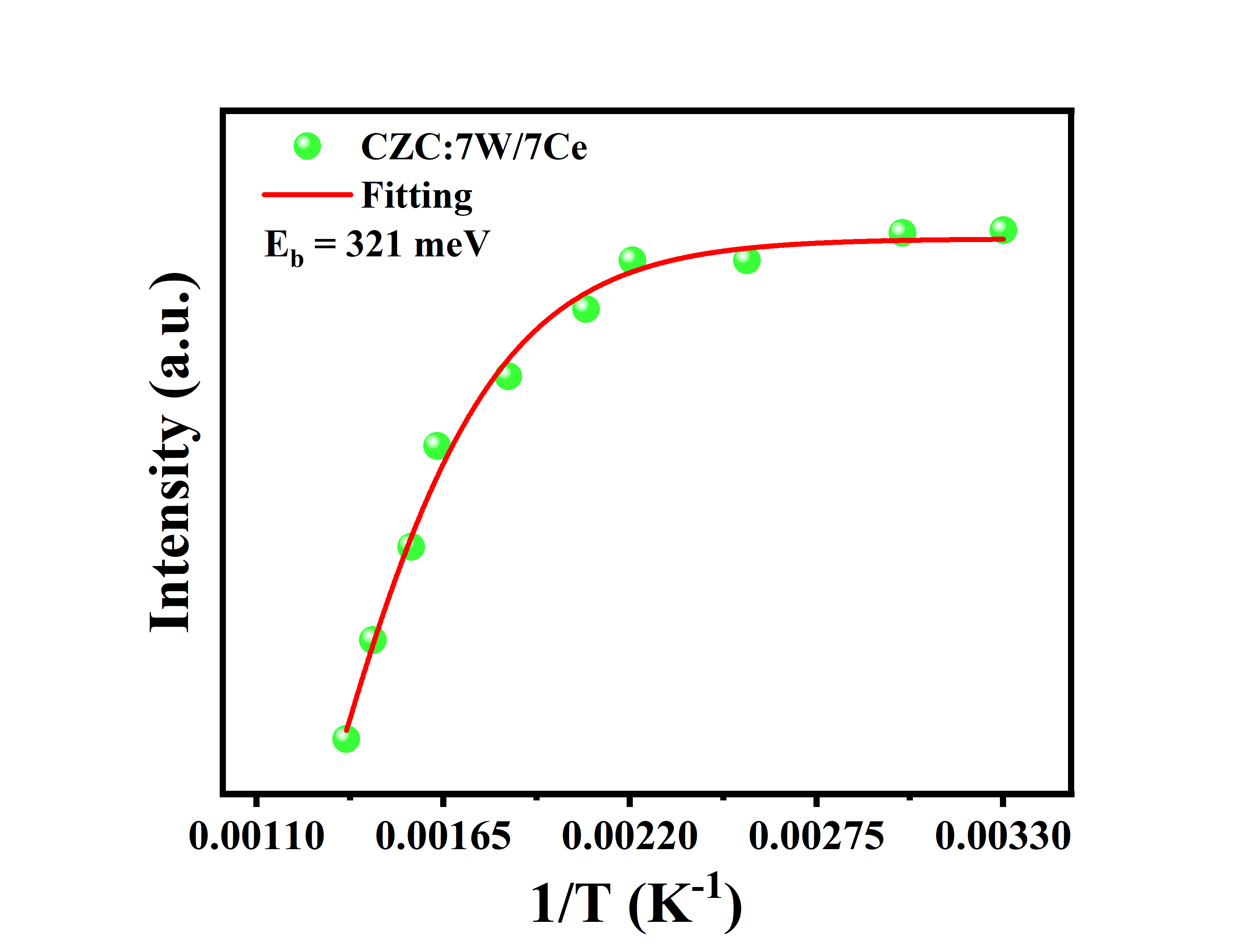


**Figure S13**. The integrated PL intensity versus 1/T and the fitting result of *E_b_* for CTC:7W/7Ce.

**Figure S14**. Electronic band structures of CZC:7W and CZC:7W/7Ce, respectively.

**Figure S15**. a) PL and PLE spectra of CZC and CZC:7W in the visible region under 254 nm excitation. b, c) PL decay times of CZC and CZC:7W at 430 nm under 254 nm excitation at different temperatures.

**Figure S16**. Temperature-dependent bandwidth of CZC:7W and Calculated *S_r_* and *S_a_* values in the 303–733 K range. Temperature-dependent bandwidth of CZC:7W/7Ce and Calculated *S_r_* and *S_a_* values in the 80–733 K range.

**Figure S17**. a) The setup of NIR spectroscopy-type temperature sensor for monitoring the temperature of autoclave using our material and commercial infrared thermal imager. b, c) The temperatures determined by both experiment and thermal imager with the cooling time.

**Figure S18**. The response and LOD values under different HF concentrations at 333, 363 and 393 K in CZC:7W/7Ce, respectively. The error bars represent the standard deviation of both response and LOD values under 5 repeated measurements.

**Table S1**. Inductively coupled plasma optical emission spectrometer (ICP-OES) data of CZC:xW/xCe (x = 4, 5, 6, 7).

**Table S2**. Performance comparison of CZC:7W and CZC:7W/7Ce with some reported broadband NIR phosphors.

**Table S3**. Bond lengths and angles for CZC.

**Table S4**. Bond lengths and angles for CZC/W.

**Table S5**. Bond lengths and angles for CZC/W/Ce.

**Table S6**. The fitting results of PL decay time for CZC (τ_0_) and CZC:7W (τ_1_) monitored at 430 nm under 254 nm excitation for different temperatures. *η* is the energy transfer efficiency from STE of [ZrCl_6_]^2–^ to [WCl_6_]^2–^ emitting center.

**Table S7**. Bond length quadratic elongation (λ_oct_) and out-of-center distortion (Δd) values for the octahedral geometry in CZC:7W and CZC:7W/7Ce.

**Table S8**. The fitting results of PL decay time for CZC:7W at 900 nm under 330 nm excitation at both low and high temperatures.

**Table S9**. The calculated adsorption energy of HF molecule on the surface of CZC and CZC:7W, respectively.

**References**

[1] G. Kresse, J. Furthmüller, *Comput. Mater. Sci.* **1996**, *6*, 15.

[2] G. Kresse, J. Furthmüller, *Phys. Rev. B* **1996**, *54*, 11169.

[3] J. P. Perdew, K. Burke, M. Ernzerhof, *Phys. Rev. Lett.* **1996**, *77*, 3865.

[4] G. Kresse, D. Joubert, *Phys. Rev. B* **1999**, *59*, 1758.

[5] P. Blöchl, *Phys. Rev. B* **1994**, *50*, 17953.

[6] H. J. Monkhorst, J. D. Pack, *Phys. Rev. B* **1976**, *13*, 5188.

[7] S. Grimme, J. Antony, S. Ehrlich, H. Krieg, *J Chem. Phys.* **2010**, *132*, 154104.

[8] S. Grimme, S. Ehrlich, L. Goerigk, *J. Comp. Chem*. **2011**, *32*, 1456.

[9] W. Huang, J. Zhang, J. Fan, P. Chen, Q. Pang, L. Zhou, *Inorg. Chem*. **2023**, *62*, 13370.

[10] X. Lu, Y. Gao, J. Chen, M. Tan, J. Qiu, *ACS Appl. Mater Interfaces* **2023**, *15*, 39472.

[11] C.-J. Tang, B.-M. Liu, L. Huang, J. Wang, Q. Tang, *J. Mater. Chem. C* **2022**, *10*, 18234.

[12] L. Yuan, Y. Jin, H. Wu, K. Deng, B. Qu, L. Chen, Y. Hu, R.-S. Liu, *ACS Appl. Mater Interfaces* **2022**, *14*, 4265.

[13] C. Wang, J. Lin, X. Zhang, H. Dong, M. Wen, S. Zhao, S. Yuan, D. Zhu, F. Wu, Z. Mu, *J. Alloys Compd.* **2023**, *942*, 168893.

[14] W. Zhou, J. Luo, J. Fan, H. Pan, S. Zeng, L. Zhou, Q. Pang, X. Zhang, *Ceram. Int*. **2021**, *47*, 25343.

[15] T. Yu, H. Sheng, S. Chen, J. Yuan, T. Deng, M. Wu, Y. Guo, Q. Zeng, *J. Am. Ceram. Soc.* **2022**, *105*, 3403.

[16] Q. Zhang, D. Liu, P. Dang, H. Lian, G. Li, J. Lin, *Laser Photonics Rev.* **2021**, *16*, 2100459.

[17] G. Liu, M. S. Molokeev, Z. Xia, *Chem. Mater.* **2022**, *34*, 1376.

[18] L. Yao, Q. Shao, M. Shi, T. Shang, Y. Dong, C. Liang, J. He, J. Jiang, *Adv. Optical Mater.* **2021**, *10*, 2102229.

[19] Q. Fan, J. Li, J. Yang, Y. Zhou, Q. Zhou, Z. Wang. *Mater. Res. Bull.* **2023**, *158*, 112065.

[20] J. Wang, X. Han, Y. Zhou, Z. Wu, D. Liu, C. Zeng, S. Cao, B. Zou. *J. Phys. Chem. Lett.* **2023**, *14*, 1371.

[21] Z. Wu, X. Han, J. Wang, Y. Zhou, K. Xing, S. Cao, J. Zhao, B. Zou, R. Zeng. *J. Mater. Chem. C* **2022**, *10*, 10292.
